# The monoterpene 1,8-cineole prevents cerebral edema in a murine model of severe malaria

**DOI:** 10.1371/journal.pone.0268347

**Published:** 2022-05-12

**Authors:** Edgleyson C. dos Santos, Leandro S. Silva, Alessandro S. Pinheiro, Douglas E. Teixeira, Diogo B. Peruchetti, Rodrigo P. Silva-Aguiar, Camila H. C. Wendt, Kildare R. Miranda, Andrelina N. Coelho-de-Souza, José Henrique Leal-Cardoso, Celso Caruso-Neves, Ana Acacia S. Pinheiro

**Affiliations:** 1 Instituto de Biofísica Carlos Chagas Filho, Universidade Federal do Rio de Janeiro, Rio de Janeiro, Brazil; 2 Instituto de Ciências Biomédicas, Universidade Estadual do Ceará, Fortaleza, Brazil; 3 Centro Nacional de Biologia Estrutural e Bioimagem, Universidade Federal do Rio de Janeiro, Rio de Janeiro, Brazil; 4 Instituto Nacional de Ciência e Tecnologia em Biologia Estrutural e Bioimagem, Conselho Nacional de Desenvolvimento Científico e Tecnológico, Rio de Janeiro, Brazil; 5 Instituto Nacional de Ciência e Tecnologia em Medicina Regenerativa, Conselho Nacional de Desenvolvimento Científico e Tecnológico, Rio de Janeiro, Brazil; 6 Rio de Janeiro Innovation Network in Nanosystems for Health, Rio de Janeiro, Brazil; Institute of medical research and medicinal plant studies, CAMEROON

## Abstract

1,8-Cineole is a naturally occurring compound found in essential oils of different plants and has well-known anti-inflammatory and antimicrobial activities. In the present work, we aimed to investigate its potential antimalarial effect, using the following experimental models: (1) the erythrocytic cycle of *Plasmodium falciparum*; (2) an adhesion assay using brain microvascular endothelial cells; and (3) an experimental cerebral malaria animal model induced by *Plasmodium berghei* ANKA infection in susceptible mice. Using the erythrocytic cycle of *Plasmodium falciparum*, we characterized the schizonticidal effect of 1,8-cineole. This compound decreased parasitemia in a dose-dependent manner with a half maximal inhibitory concentration of 1045.53 ± 63.30 μM. The inhibitory effect of 972 μM 1,8-cineole was irreversible and independent of parasitemia. Moreover, 1,8-cineole reduced the progression of intracellular development of the parasite over 2 cycles, inducing important morphological changes. Ultrastructure analysis revealed a massive loss of integrity of endomembranes and hemozoin crystals in infected erythrocytes treated with 1,8-cineole. The monoterpene reduced the adhesion index of infected erythrocytes to brain microvascular endothelial cells by 60%. Using the experimental cerebral malaria model, treatment of infected mice for 6 consecutive days with 100 mg/kg/day 1,8-cineole reduced cerebral edema with a 50% reduction in parasitemia. Our data suggest a potential antimalarial effect of 1,8-cineole with an impact on the parasite erythrocytic cycle and severe disease.

## Introduction

Malaria is a life-threatening parasitic disease and a major public health problem. Annually, about 229 million cases and 409,000 deaths are registered worldwide [1]. The most severe form of the disease is attributed to *Plasmodium falciparum* infection whereby infected erythrocytes sequester in the microvasculature of critical organs inducing different pathologies, including acute kidney injury, acute respiratory distress syndrome, and cerebral malaria [2–4]. The use of artemisinin-based combination therapies (ACTs) as well as the increased use of bed nets, mosquito vector control, and introduction of rapid diagnostic tests, resulted in an estimated 37% reduction in the number of malaria-induced deaths [5]. However, the remarkable ability of *Plasmodium* species to become resistant to the existing front-line drugs threatens to reverse these fragile gains [5, 6]. This challenge highlights the necessity for the continued search for new antimalarials.

Malaria symptoms occur during the erythrocytic cycle of the disease, characterized by the infection of circulating erythrocytes in the host. After infection, the parasite develops inside the red blood cells, giving rise to different intracellular forms until they reach the mature state called schizont. Subsequently, schizonts generate numerous merozoites, which are released into the blood on the rupture of the red blood cells (RBCs). Merozoites quickly infect new cells, perpetuating the erythrocytic cycle and increasing blood parasitemia [3, 7]. Thus, control of the erythrocytic cycle reduces infection and consequently the evolution of the disease to the most severe forms [8].

Naturally occurring compounds such as essential oils have emerged as medicinal agents because of their capacity to remediate diverse diseases and infections [9, 10]. 1,8-cineole is an oxygenated monoterpene, also known as eucalyptol [11]. It is a major compound in the essential oils of many plants, including *Artemisia annua*, which, historically, has been widely used to treat fever and malaria [12]. In general, terpenes have confirmed inhibitory activity against *P*. *falciparum* [13, 14], however, there are only a few studies on the antiplasmodial activity of 1,8-cineol in the literature. As an isolated compound, it was postulated that 1,8-cineole reduced *P*. *falciparum* parasitemia in vitro [15], but how it correlates to the development of severe diseases, such as cerebral edema, remains to be determined. Moreover, the anti-inflammatory activity of the monoterpene has been described [11, 16, 17]. In the present study, using the erythrocytic cycle of *P*. *falciparum*, we demonstrate that 1,8-cineole reduces parasitemia and intracellular development of the parasite. Moreover, we provide evidence that the monoterpene partially prevents cerebral edema in an experimental cerebral malaria (ECM) model. These results could contribute to the development of a new class of antimalarials that focus on the erythrocytic cycle of the parasite.

## Materials and methods

### Drugs

1,8-cineole (99% purity), d-sorbitol, Evans blue, glucose, HEPES, magnesium chloride, potassium chloride, EGTA, PIPES, hypoxanthine, glutaraldehyde, osmium tetroxide, potassium ferrocyanide, lead citrate, ethanol, formamide, M199 medium, and sodium bicarbonate were obtained from Sigma-Aldrich (St. Louis, MO, USA). Artesunate (#27354) was obtained from Chengdu Okay Pharmaceutical Co. (Chengdu, Sichuan, China). l-Glutamine, gentamicin, penicillin/streptomycin, RPMI 1640, fetal bovine serum, phosphate-buffered saline, and sodium pyruvate were obtained from Thermo Fisher Scientific (Waltham, MA, USA). DMSO, sucrose and prepared formaldehyde were purchased from Isofar (Duque de Caxias, RJ, Brazil). Gas mixture (5% CO_2_, 5% O_2_, and 90% N_2_) was obtained from White Martins Gases Industriais (Rio de Janeiro, RJ, Brazil). A kit for hematologic staining (#LB170117) was purchased from Laborclin (Pinhais, PR, Brazil). LDH liquiform (#86) was obtained from Labtest Diagnostica (Lagoa Santa, MG, Brazil). GraphPad Prism software (version 8.0, GraphPad Software, San Diego, CA, USA, www.graphpad.com) was used for the statistical analysis.

### Ethics statement

Parasite cultures were supplemented with A+-type blood samples collected from healthy volunteers, randomly selected, who provided written informed consent. All procedures were designed and approved by the Research Ethics Committee of the Hospital Universitário Clementino Fraga Filho from the Federal University of Rio de Janeiro (permit number 074/10).

The ECM model was used to determine the potential antimalarial effect of 1,8-cineole on peripheral blood parasitemia and cerebral edema. This study was performed according to the Guide for the Care and Use of Laboratory Animals of the National Institutes of Health. All experimental protocols were assessed and approved by the Institutional Ethics Committee of Federal University of Rio de Janeiro (UFRJ) (number 041/18). All surgery was performed under anesthesia using a ketamine/xylazine mixture, and all efforts were made to minimize suffering.

### *Plasmodium falciparum* erythrocytic cycle

The cultures of *P*. *falciparum* were performed as described [18–21]. *P*. *falciparum* (W2 strain) were kept in vitro in RPMI 1640 medium supplemented with 50 μg/mL gentamicin and 10% A+-type human plasma at 5% hematocrit in 25 cm^2^ flasks. Parasite cultures were incubated in a gas-controlled atmosphere (5% CO_2_, 5% O_2_, and 90% N_2_) for at least 24 h. Parasitemia was evaluated daily in hematologic stained thin blood smears by optical microscopy. Parasitemia was expressed by the percentage of infected erythrocytes.

### Parasite culture synchronization

Parasite culture synchronization was carried out as published previously [18–21]. Briefly, the synchronization process involved treating infected erythrocytes with 5% d-sorbitol for 10 min to eliminate the cells infected with mature forms. After treatment, cells were washed and recultured to allow the formation of new schizonts. The effects of 1,8-cineole were tested in cultures containing 1% parasitemia. DMSO (at final concentrations <1%) was used as a vehicle for 1,8-cineole.

### Treatment with 1,8-cineole and determination of the half maximal inhibitory concentration

The infected erythrocytes were treated with different concentrations of 1,8-cineole (65–6483 μM) for 24 h. After incubation, the percentage of parasite ring forms was determined in hematologic stained thin blood smears using optical microscopy. Then, the half maximal inhibitory concentration (IC_50_) for the effect of 1,8-cineole was calculated by nonlinear regression analysis with the best fit of the experimental values using GraphPad Prism software. It was assumed that the dose-response curve has a standard slope, equal to a Hill slope of 1. Three independent experiments were performed in triplicate.

### Hemolysis assay

The hemolysis assay was performed as described previously [18]. Non-infected erythrocytes were incubated or not with 1,8-cineole (65–3241 μM) for 24 h. DMSO at a final concentration of 0.5% was used as a vehicle for 1,8-cineole. Then, the cell supernatants were collected and clarified by centrifugation at 600 × *g* for 8 min to measure free hemoglobin spectrophotometrically at 530 nm. The results are expressed as a percentage of a positive control.

### Analysis of the ultrastructure by transmission electron microscopy

Transmission electron microscopy was performed as published previously [18]. Briefly, infected erythrocytes (1% parasitemia, 5% hematocrit, enriched in mature forms) were treated or not with 972 μM 1,8-cineole for 48 h under the same culture conditions described for the *P*. *falciparum* erythrocytic cycle. Then, cultures were washed twice in 0.1 M PHEM buffer (30 mM PIPES, 10 mM HEPES, 5 mM EGTA, 2.5 mM MgCl_2_, 35 mM KCl [pH 7.2]) and fixed for 24 h in a mixture containing 2.5% glutaraldehyde, 4% sucrose, and 4% freshly prepared formaldehyde in 0.1 M PHEM buffer (pH 7.2). After washing in 0.1 M PHEM buffer, samples were post-fixed in 1% osmium tetroxide plus 0.8% potassium ferrocyanide in 0.1 M cacodylate buffer for 40 min, dehydrated in ethanol, and embedded in epoxide resin. Ultrathin sections (70 nm) were cut and stained for 20 min in 5% aqueous uranyl acetate and 5 min in lead citrate. Samples were observed in a Tecnai-Spirit transmission electron microscope (Thermo Scientific) operating at 120 kV. Images were obtained with a 2 k Veleta camera (Olympus).

### Culture of brain microvascular endothelial cells

Brain microvascular endothelial cells (BMECs), an immortalized brain microvascular endothelial cell line of human origin, originally used as a model of the blood-brain barrier [21, 22], were maintained in medium 199 (M199, Sigma-Aldrich) supplemented with 10% heat-inactivated fetal calf serum (Invitrogen, Carlsbad, CA, USA) and 1% penicillin/streptomycin (Sigma Chem Co, St Louis, MO, USA) at 37°C in 5% CO_2_.

### Adhesion assay

We used an adhesion assay to evaluate the ability of infected erythrocytes to adhere to endothelial cells. BMECs were plated in 24-well culture chambers (5 × 10^4^ cells/well) and cultured for 24 h in M199 medium supplemented with 10% fetal calf serum (pH 7.4). Non-infected RBCs or *P*. *falciparum*-infected erythrocytes (iRBC, 4 × 10^5^ cells/well, 5% parasitemia) were incubated or not with 972 μM 1,8-cineole for 2 h before coculture with BMEC for an additional 2 h under the same culture conditions as for BMECs. Non-adherent erythrocytes were washed out with phosphate-buffered saline, and the adhered cells were fixed and stained with hematologic staining. The number of adhered erythrocytes per BMEC was determined by direct counting using optical microscopy (10 fields/well). The data obtained were used to calculate the adhesion index: adhesion index = {[(BMECs with bound erythrocytes)/total number of BMECs] × [(erythrocytes bound to BMECs)/total number of BMECs]} × 100.

### Measurement of lactate dehydrogenase

Lactate dehydrogenase (LDH) activity in the cell supernatant of BMECs was detected as described previously [18]. BMECs were incubated or not with different concentrations of 1,8-cineole (ranging from 324 to 3241 μM). Then, the cell supernatant was collected and LDH activity was measured by a kinetic-UV (pyruvate-lactate) method using a commercial kit purchased from Labtest. The results are expressed as the percentage of a positive control (LDH activity in the lysate of BMECs obtained by incubation of cells with 1% Triton X-100).

### Animals and experimental cerebral malaria

Male C57BL/6 mice (8–12 weeks) were provided by the Animal Care Facility of the Health Science Center of the UFRJ. The animals were kept in cages in a temperature-controlled room (22°C–24°C) with a 12 h light/dark cycle, with access to food and water ad libitum.

ECM was induced as published previously [18, 23–27]. Briefly, malaria infection was induced by intraperitoneal injection of 1 × 10^6^ *P*. *berghei* ANKA (PbA)-infected erythrocytes in normal C57BL/6 mice. Peripheral blood parasitemia was determined daily in hematologic stained thin blood smears using optical microscopy. The results are expressed as the percentage of infected cells. Mice were divided into **5** groups: (1) control, non-infected mice; (2) PbA-infected mice; (3) non-infected mice treated with 1,8-cineole; (4) PbA-infected mice treated with 1,8-cineole; and (5) PbA-infected mice treated with artesunate. The treatment with 1,8-cineole (100 mg/kg/day) or artesunate (10 mg/kg/day) started on the day of infection using daily doses of via intraperitoneal injection for 6 consecutive days. Peripheral blood parasitemia was assessed on days 3, 4, 5 and 6 post-infection, and cerebral edema was assessed at the end of the experiment.

### Cerebral edema

The evaluation of cerebral edema was performed using the Evans blue extravasation assay as described previously [18, 25]. Briefly, mice received an intravenous injection of 1% Evans blue dye solution. After 1 h, the mice were euthanized and their brain was removed, weighed, and incubated in 2 mL of formamide (37°C, 48 h) to extract the dye. Absorbance of the supernatant was measured at 620 nm. The concentration of dye was determined using a standard curve. The data are expressed as milligrams of dye normalized per gram of tissue.

### Statistical analysis

All results are expressed as the mean ± standard deviation (SD) of at least 3 independent experiments. GraphPad Prism 8 was used for the statistical analysis. Comparison of the different experimental groups was determined by one-way analysis of variance (ANOVA), followed by the Tukey post-test. Significance was determined as a *P* value <0.05.

## Results

### 1,8-cineole reduces *Plasmodium falciparum* parasitemia *in vitro*

**Fig 1:**
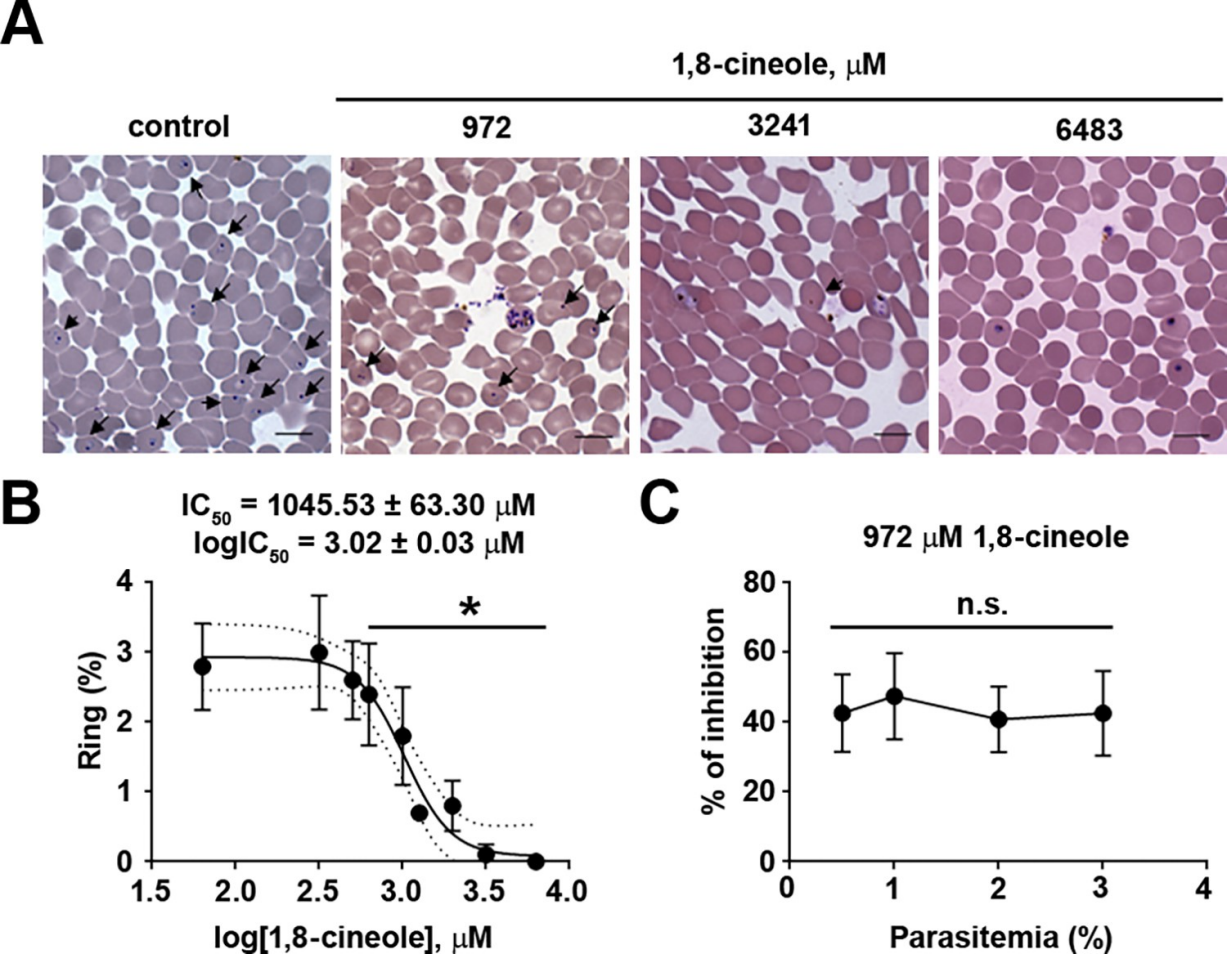
The monoterpene 1,8-cineole decreased parasitemia levels in vitro. Synchronized cultures of erythrocytes infected with mature forms of *P*. *falciparum* were incubated with 1,8-cineole for 24 h to monitor parasitemia. (A and B) Dose-response effect of 1,8-cineole on parasitemia (*n* = 4). (A) Representative images of the different experimental conditions. Arrows indicate infected erythrocytes with ring form. (B) Infected erythrocytes (1% parasitemia, 3%–5% hematocrit) were incubated for 24 h with increasing concentrations of 1,8-cineole (65–6483 μM). After incubation, the percentage of ring forms was determined by optical microscopy as described in the Materials and methods section. (C) The inhibitory effect of 1,8-cineole was not dependent on the level of parasitemia (*n* = 5). The number of rings was monitored after treating erythrocytes infected with increasing amounts of schizonts (0.5%–3% parasitemia) with 972 μM 1,8-cineole for 24 h. The results are presented as the mean ± SD. *P < 0.05 versus control. n.s., not significant.

In the first experimental group, we evaluated the effect of the monoterpene 1,8-cineole in the erythrocytic cycle of *P*. *falciparum*. For this, infected erythrocytes enriched in the schizont form (1% parasitemia, 3%–5% hematocrit) were incubated with increasing concentrations of 1,8-cineole from 65 to 6483 μM (corresponding to 10–1000 μg/mL) and parasitemia, expressed as the percentage of rings (% ring), was assessed after 24 h. This compound decreased the % ring in a dose-dependent manner with an IC_50_ of 1045.53 ± 63.30 μM (or 150 μg/mL), and it reached 100% inhibition at 6483 μM (Fig 1A and 1B). The decrease in parasitemia levels was not due to any hemolytic effect, because the levels of hemoglobin detected in the supernatant of healthy erythrocytes at all concentrations of 1,8-cineole tested was less than 10% and similar to the that of the controls (RPMI and the vehicle DMSO) (S1 Fig).

To determine whether the inhibitory effect of the monoterpene was observed using different levels of parasitemia, we tested the effect of 1,8-cineole at the IC_50_ concentration (972 μM) in cultures with parasitemia ranging from 0.5% to 3%. We detected 40% inhibition regardless of the level of parasitemia (Fig 1C).

### 1,8-cineole treatment irreversibly affects intracellular development of *P*. *falciparum*

**Fig 2:**
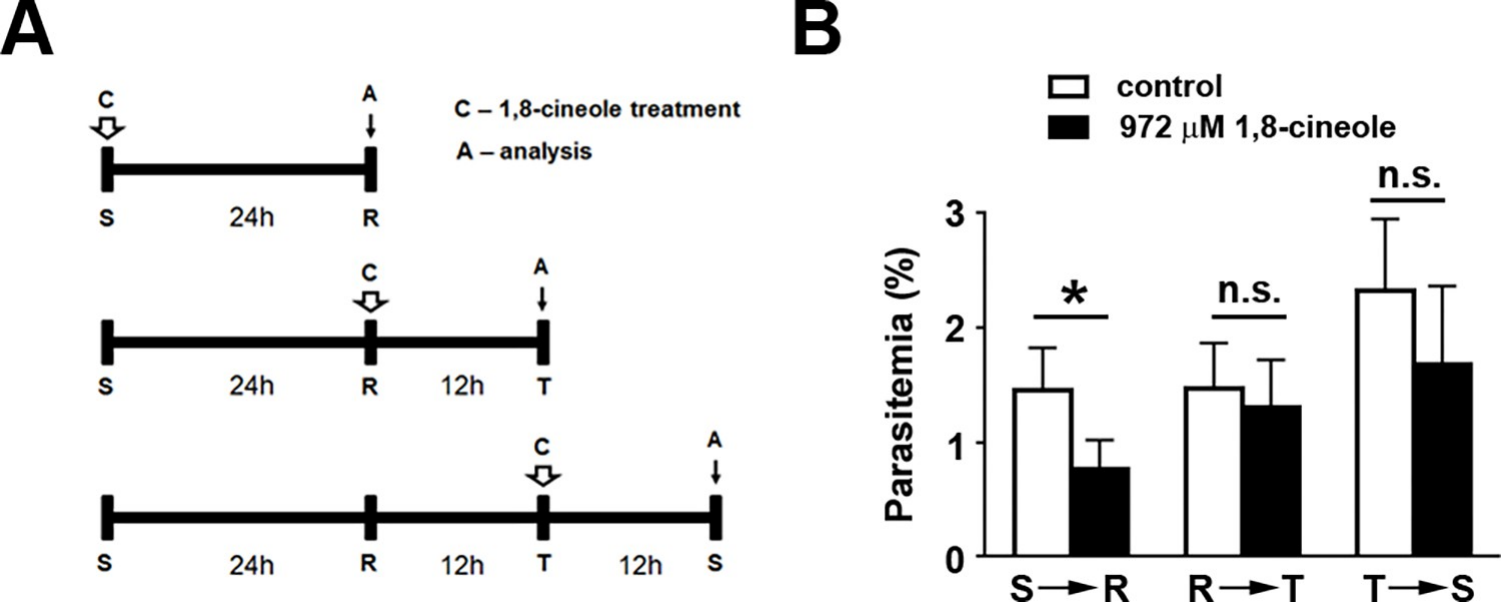
Assessment of the effect of 1,8-cineole on specific stages of the *P*. *falciparum* life cycle and evolution. (A) Experimental design. Different evolutive forms of *P*. *falciparum* were treated with 972 μM 1,8-cineole, and parasitemia was determined by optical microscopy (analysis). The arrows indicate when treatment with 1,8-cineole started. S, schizonts; R, rings; T, trophozoites. (B) Determination of parasitemia right after differentiation (*n* = 5). S → R, evolution from schizont to ring; R → T, evolution from ring to trophozoite; T → S, evolution from trophozoite to schizont. The results are presented as the mean ± SD. *P < 0.05 versus control. n.s., not significant.

We observed a decrease in the percentage of parasitemia in the presence of 1,8-cineole, therefore we decided to characterize whether the monoterpene affects the intracellular development of the parasite. First, we aimed to identify which parasite form was more susceptible to the treatment. For this, synchronized cultures of *P*. *falciparum* enriched in schizonts, rings, or trophozoites (1% parasitemia) were treated or not with 972 μM 1,8-cineole, and parasitemia was determined right after transition to the next parasite form (Fig 2A). Under the experimental conditions used, parasitemia levels were not significantly changed when rings or trophozoites were treated with the compound. However, the treatment of schizonts produced 40% inhibition, characterizing a possible schizonticide effect of 1,8-cineole (Fig 2B).

**Fig 3:**
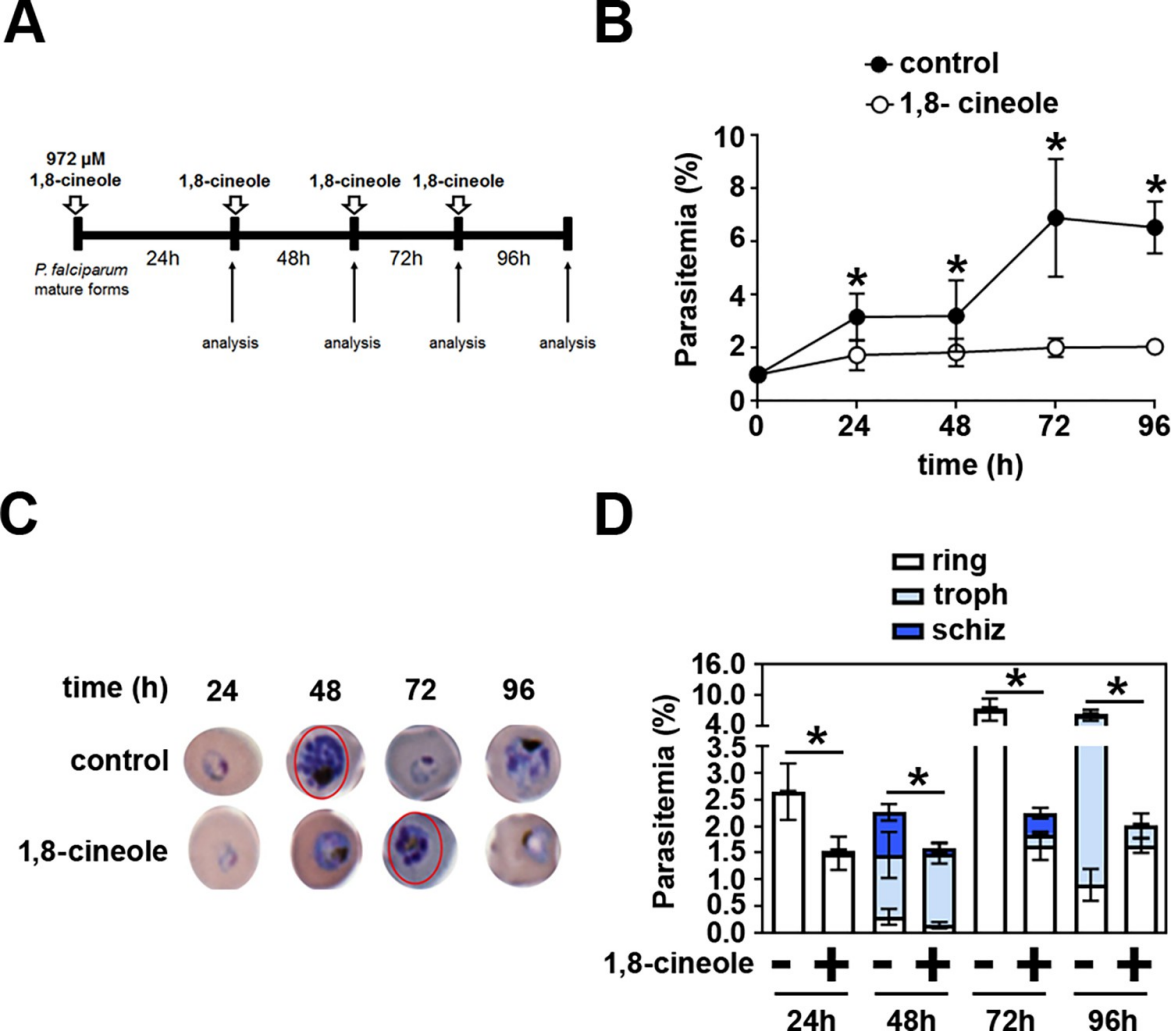
Treatment with 1,8-cineole impaired the intracellular development of *Plasmodium falciparum*. (A) Experimental design. Non-synchronized cultures of *P*. *falciparum* (1% parasitemia) were treated daily (white arrows) or not with 972 μM 1,8-cineole for 96 h. Parasitemia was determined by optical microscopy every 24 h (analysis). (B) Assessment of parasitemia (*n* = 6). (C) Representative images showing that treatment with 1,8-cineole arrests intracellular development of the parasite. (D) Assessment of the distribution of evolutive forms of the parasite in the presence of 972 μM 1,8-cineole (*n* = 6). Troph, trophozoite; schiz, schizont. The results are presented as the mean ± SD. *P < 0.05 versus control.

In the next step, 1,8-cineole was added to non-synchronized cultures of *P*. *falciparum* (1% parasitemia) daily for 4 consecutive days to evaluate intracellular development of the parasite during 2 parasite cycles (Fig 3A). Parasite growth proceeded normally under the control conditions, achieving 7% parasitemia after 96 h, but in the presence of 972 μM 1,8-cineole, the number of rings remained at low levels, around 1.5%, throughout the experiment (Fig 3B). Optical microscopy revealed important morphological changes, especially in mature trophozoites and schizonts (Fig 3C). Under the control situation, parasites progressed to mature forms at 48 h in culture (Fig 3C), whereas with 1,8-cineole treatment, at the same time point, schizonts were strikingly reduced in size, suggesting a delay in the intraerythrocytic progression of the parasite; this became more evident in a second development cycle. After 96 h, parasites appear smaller in size, with less formation of hemozoin (Fig 3C). Moreover, these morphological changes are associated with reduction in the percentage of parasitemia. Because ring formation is reduced, this has an impact on the progression of the parasite cycle (Fig 3D).

**Fig 4:**
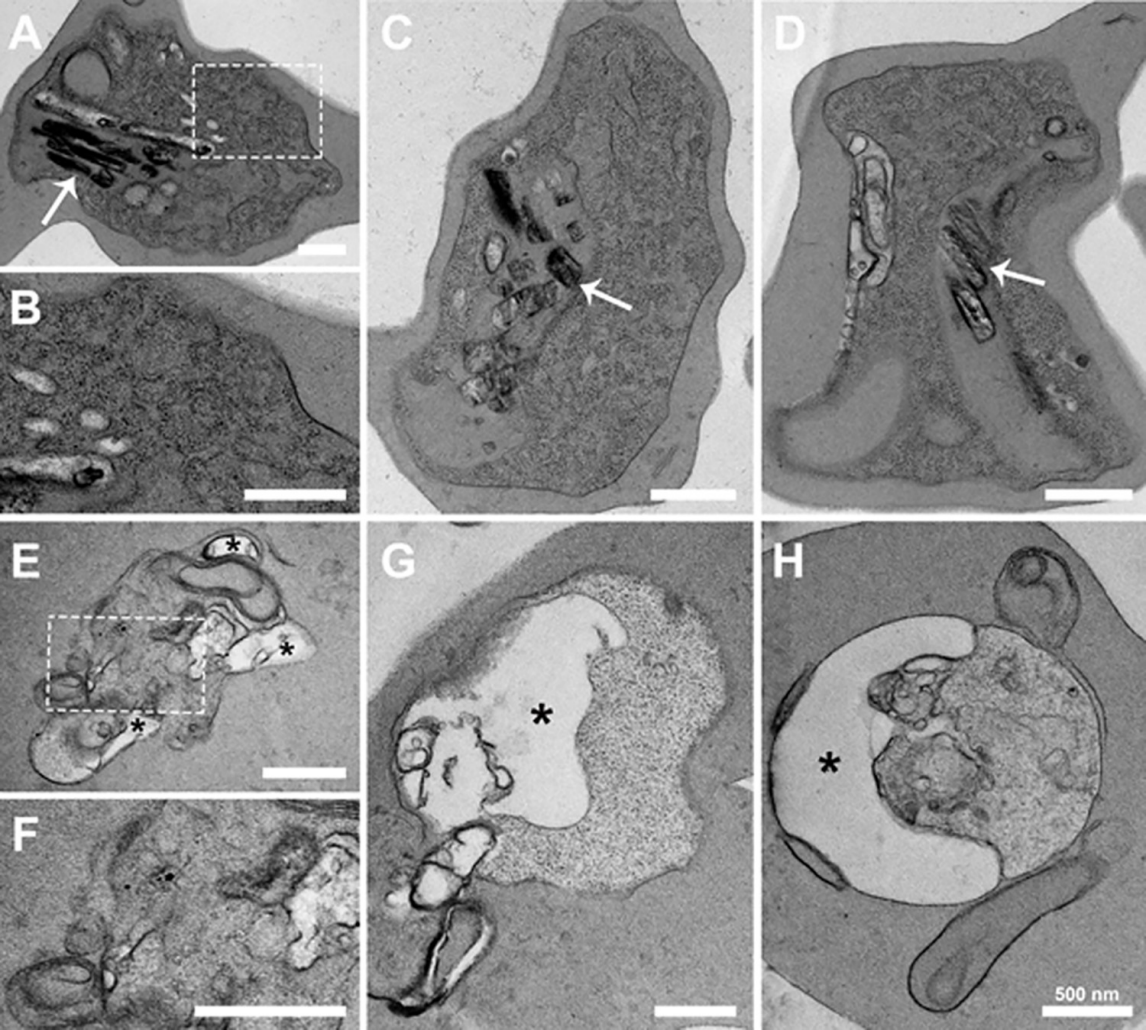
Treatment with 1,8-cineole induced ultrastructural changes in *Plasmodium falciparum*. Control cells (A–D) showed the characteristic structure of trophozoites, including an extensive endoplasmic reticulum network (A, white rectangle; B) and digestive vacuoles filled with hemozoin crystals (A, C, and D arrows). In contrast, cells incubated with 972 μM 1,8-cineole showed loss of endomembrane integrity (E, white rectangle; F) and empty vacuoles resembling residual food vacuoles (E, G, and H, asterisks).

Electron microscopy analysis of cells incubated with 1,8-cineol showed several structural changes compared with control cells (Fig 4). Control trophozoites (Fig 4A–4D) exhibited a normal aspect, showing a well-preserved and elaborated endomembrane system, including the endoplasmic reticulum network spread throughout the cell cytoplasm (Fig 4A, white rectangle; Fig 4B) and large digestive vacuoles filled with hemozoin crystals (Fig 4A, 4C, and 4D, white arrows). In contrast, parasites incubated with 972 μM 1,8-cineole (Fig 4E–4H) did not show the characteristic endoplasmic reticulum network (Fig 4E, white rectangle; Fig 4F) or hemozoin crystals (Fig 4E, 4G, and 4H), suggesting a massive loss of membrane integrity that may lead to cell death. Empty vacuoles, most likely residual food vacuoles (Fig 4E, 4G, and 4H, asterisks) were also observed in treated cells, in some cases occupying a large area of the cytoplasm.

**Fig 5:**
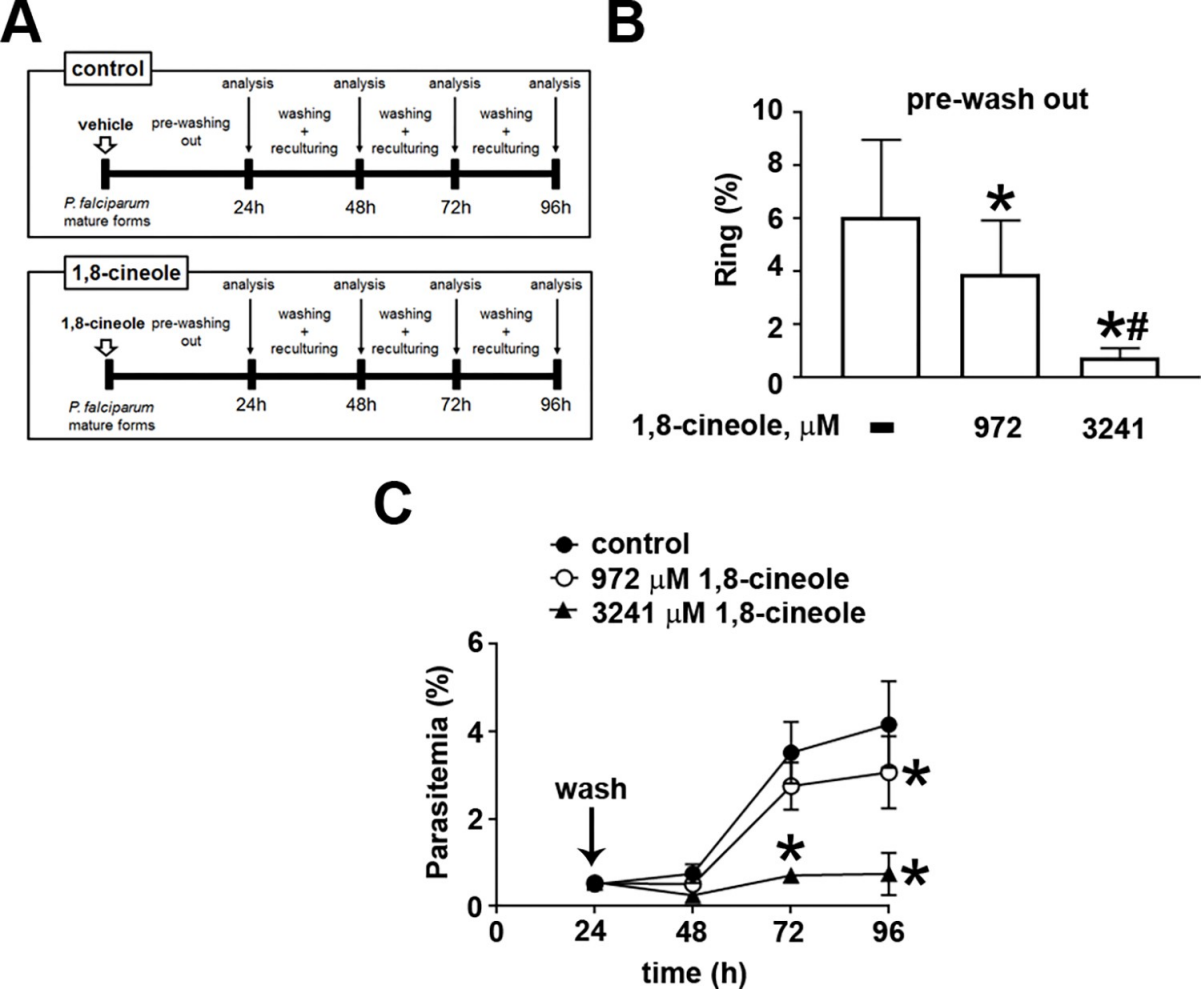
The inhibitory effect of 1,8-cineole on the erythrocytic cycle of *Plasmodium falciparum* is not reversible. (A) Experimental design. Synchronized cultures of erythrocytes infected with *P*. *falciparum* schizonts were incubated or not with 972 or 3241 μM 1,8-cineole (white arrows) for 24 h. Cells were harvested, and parasitemia was determined by optical microscopy (analysis). The cells were then washed, and parasitemia was adjusted to 0.5% before reculturing them in absence of the compound for an additional 3 consecutive days. Parasitemia was determined every 24 h (analysis at 24, 48, 72, and 96 h after the first washing). (B) The inhibitory effect of 1,8-cineole on parasitemia levels in the first 24 h, before washing out the compound (*n* = 3). (C) The effect of the time course of 1,8-cineole on parasitemia after washing out the compound from the cells (*n* = 3). The results are presented as the mean ± SD. *P < 0.05 versus control, #*P* < 0.05 versus 972 μM 1,8-cineole.

To evaluate whether 1,8-cineole-induced inhibition of ring formation was a reversible process, schizont cultures (1% parasitemia) were treated with 972 or 3241 μM 1,8-cineole for 24 h. After incubation, cells were washed to remove the compound and recultured under normal culture conditions, with parasitemia adjusted to 0.5% in both groups, for an additional 72 h (Fig 5A). In the first 24 h of treatment, before washing the cells, treatment with 972 μM 1,8-cineole reduced ring formation by about 40% as expected. A more pronounced inhibition was observed when cultures were treated with 3241 μM 1,8-cineole, achieving 85% inhibition (Fig 5B). After washing out the compound, and under the control conditions, parasite differentiation and growth were observed. In the presence of 1,8-cineole, the inhibitory profile was sustained at least for the additional 72 h incubation for both concentrations used, suggesting irreversibility of the effect of 1,8-cineole (Fig 5C).

### 1,8-Cineole reduces adhesion of infected erythrocytes to brain microvascular endothelial cells

**Fig 6:**
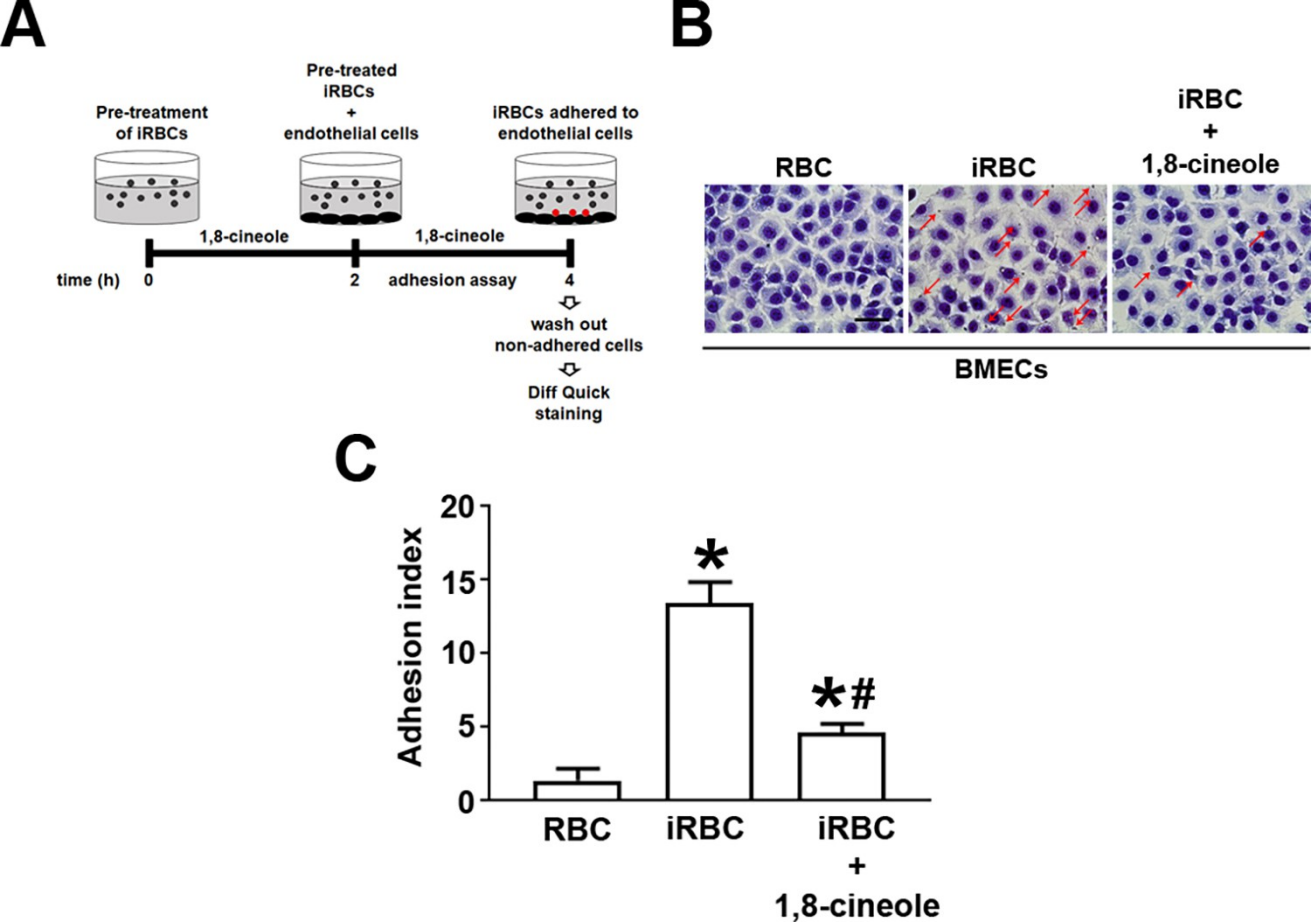
1,8-cineole inhibited the adhesion of *Plasmodium falciparum*-infected erythrocytes to brain macrovascular epithelial cell monolayers. (A) Experimental design. BMECs were cultured in 24-well plates (4 × 10^5^ cells/well) for 24 h. The erythrocytes were pre-treated with 1,8-cineole for 2 h and then 5 × 10^4^ erythrocytes/well were cocultured with BMECs for an additional 2 h (adhesion assay), generating 3 different experimental groups: (1) non-infected red blood cells (RBC, control); (2) infected red blood cells (iRBCs, 5% parasitemia); or (3) iRBCs in the presence of 972 μM 1,8-cineole. The adhesion assay was performed as described in the Materials and methods section. (B) Representative images of erythrocytes adhered to BMEC monolayers. Red arrows indicate adhered iRBCs. Scale bar, 50 μm. (C) Adhesion index. Experiments were performed in triplicate, using 3 independent cell suspensions. Results are expressed as the mean ± SD. *P < 0.05 versus RBCs, #*P* < 0.05 versus iRBCs.

The phenomenon of sequestration observed in falciparum malaria is characterized by the adhesion of *Plasmodium*-infected erythrocytes to endothelial cells lining the microvasculature of different tissues [28]. This phenomenon is a consequence of intracellular development of the parasite, and it is implicated in the pathogenesis of severe disease [28]. In the next experimental group, we verified whether the modifications induced by 1,8-cineole in infected erythrocytes could have an impact on their adhesion to endothelial cells. To test this hypothesis, *P*. *falciparum***-**infected erythrocytes were pre-treated with 972 μM 1,8-cineole for 2 h, and then cocultured with a monolayer of BMECs for an additional 2 h. The cells were then washed to remove unbound cells before determining the adhesion index as described in the Materials and methods section (Fig 6A). As expected, infected erythrocytes had a great ability to bind to BMECs compared with non-infected cells. The treatment with the monoterpene reduced the adhesion index by 60% (Fig 6B and 6C). The decrease in the adhesion index was not due to the loss of viability of the endothelial cells, because LDH activity in the supernatant of cultures treated with 1,8-cineole was around 10% (S2 Fig). These results reinforce the suggestion that 1,8-cineole is a prominent antimalarial candidate.

### 1,8-cineole attenuates severe disease in a model of experimental cerebral malaria

**Fig 7:**
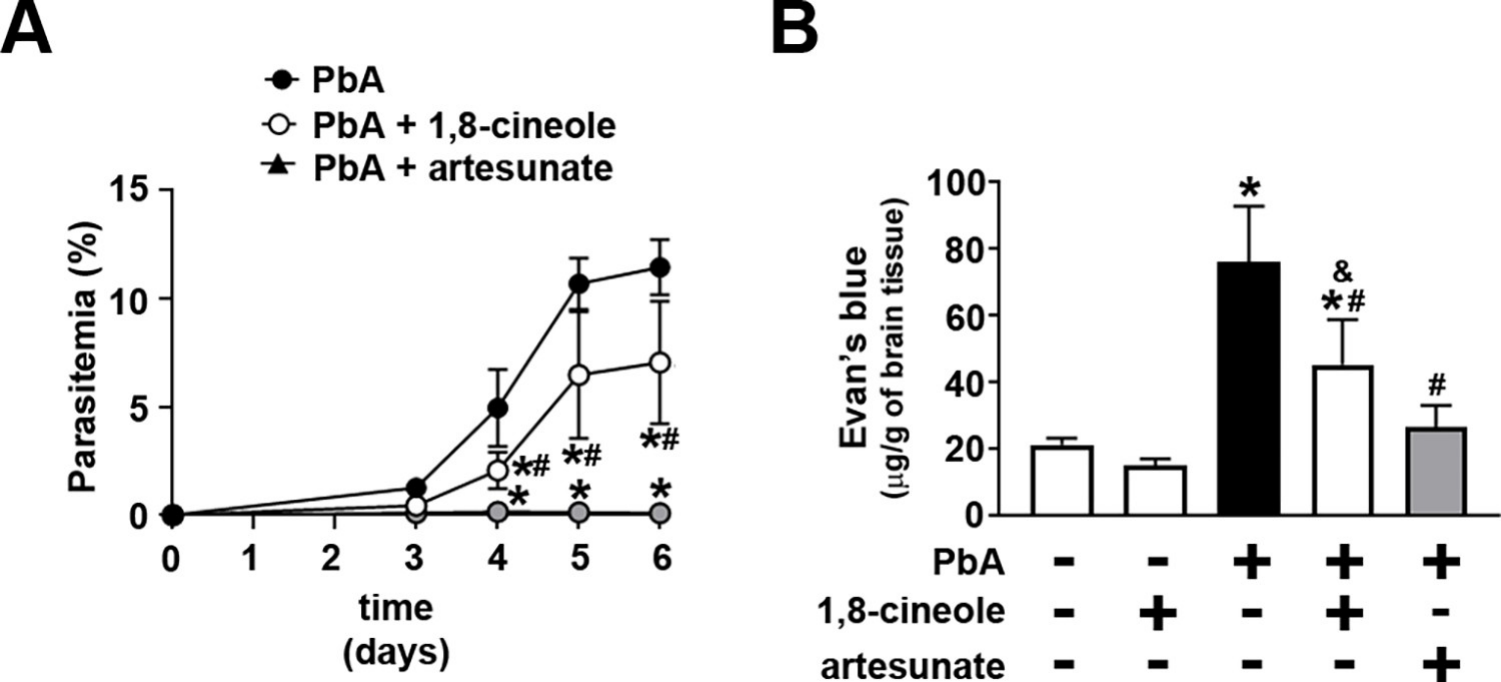
Treatment with 1,8-cineole reduced infection and cerebral edema in a model of experimental cerebral malaria. C57BL/6 mice were infected with *P*. *berghei* ANKA (PbA) before treatment with 100 mg/kg/day of 1,8-cineole or 10 mg/kg/day of artesunate via intraperitoneal injection for 6 consecutive days. (A) Peripheral blood parasitemia was determined in hematologic stained thin blood smears by optical microscopy (*n* = 5). As depicted, parasitemia was determined at days 3, 4, 5 and 6 post-infection. (B) Assessment of cerebral edema (*n* = 4). At day 6 post-infection, cerebral edema was assessed by Evans blue dye extravasation assay as described in the Materials and methods section. The results are expressed as the mean ± SD. (A) *P < 0.05 versus PbA; ^#^*P* < 0.05 versus PbA + artesunate. (B) *P < 0.05 versus control mice (non-infected mice); ^#^*P* < 0.05 versus PbA; ^&^*P* < 0.05 versus PbA + artesunate.

In the next experimental group, we used ECM to evaluate whether the inhibitory effect of 1,8-cineole on the erythrocytic cycle of the parasite is reproduced in vivo, consequently promoting a protective effect. C57BL/6 mice infected with PbA were treated or not with 100 mg/kg 1,8-cineole or 10mg/kg artesunate (intraperitoneally) daily for 6 consecutive days. Under the experimental conditions, without treatment, peripheral blood parasitemia reached almost 12% at day 6 post-infection and the mice developed cerebral edema as revealed by the Evans blue extravasation assay (Fig 7A and 7B). Treatment with 1,8-cineole partially reduced parasitemia over time, achieving 45% inhibition, while the treatment with artesunate completely abolished it (Fig 7A). Moreover, 1,8-cineole treatment partially prevented the development of cerebral edema while artesunate treatment completely avoided it (Fig 7B). It is worth to mention that treatment of non-infected mice with 1,8 cineole did not induce cerebral edema.

## Discussion

In this study, we characterized the schizonticide effect of the monoterpene 1,8-cineole using the erythrocytic cycle of *P*. *falciparum* as well as an ECM model by infecting susceptible C57BL6 mice with PbA. Our data suggest that 1,8-cineole has a potential antimalarial effect based on the following: (1) it impairs the erythrocytic cycle and the intracellular development of the parasite; (2) it inhibits adhesion of infected erythrocytes to BMECs; and (3) it protects against the development of cerebral edema. These results demonstrate the activity of 1,8-cineole against *Plasmodium* sp., suggesting its use in therapeutic strategies to treat severe disease.

In a dose-dependent experiment, we demonstrated that 1,8-cineole decreased parasitemia according to increasing concentrations of the monoterpene, with an IC_50_ of 1045.53 ± 63.30 μM (or 150 μg/mL). Su et al. [15] reported a similar IC_50_ value against the same strain of *P*. *falciparum* as used in our work. Using headspace gas chromatography to assess the real concentration of cineole found in the culture medium, the group demonstrated that the IC_50_ was more than 80% less than the calculated value, achieving a concentration that is unlikely to be toxic to the host, which makes it suitable for drug development [15]. We did not detect any hemolytic activity even in the presence of high concentrations of 1,8-cineole or toxic effects on other nucleated cells, because low levels of LDH were found in the supernatant of BMECs incubated with increasing concentrations of 1,8-cineole. In agreement, the low toxicity of 1,8-cineole has also been attested by other groups [29].

As an isolated compound, 1,8-cineole has been proposed to have antibacterial, antiviral, and antifungal activities [30–32], but studies on its effect against parasites including *Plasmodium* sp. are rare in the literature. Arrest of the growth of chloroquine-sensitive and chloroquine-resistant *P*. *falciparum* has been observed [15]. Here, we demonstrated that the monoterpene has an inhibitory effect not only on parasitemia but also on intracellular development of the parasite. The inhibitory effect of 1,8-cineole in reducing parasitemia is sustained even after washing out the monoterpene. This result could be explained in terms of the lipophilicity of the compound, perpetuating its repressive effect over time. In agreement with our findings, essential oils containing 1,8-cineole have been shown to inhibit [^3^H]hypoxanthine uptake by *P*. *falciparum*, which reflects the inhibition of parasite growth [33].

A more prominent inhibitory effect of 1,8-cineole was observed when mature forms were treated with the monoterpene, characterizing a possible schizonticide effect and consequently inhibition of early ring formation, similar to other well-known antimalarials [34, 35]. At this time point, the morphology modification revealed by optical microscopy was also relevant; the parasite was reduced in size with apparent loss of the malaria pigment, hemozoin. Ultrastructural analysis of trophozoites treated with 1,8-cineole confirmed derangement of internal membranes and the absence of crystals of hemozoin. During development, *Plasmodium* sp. degrade internalized hemoglobin, and the toxic free heme is immobilized into hemozoin as a mechanism to avoid cellular damage [36, 37]. We could not observe any crystal formation in the presence of 1,8-cineole, therefore it is possible to imagine that this mechanism to detoxify free heme is impaired by treatment with the monoterpene. However more experiments are necessary to confirm this hypothesis.

In humans, falciparum-associated pathologies seem to be dependent on parasite sequestration, due to the adhesion of infected erythrocytes to the endothelium [28]. Using an in vitro model of co-incubation, we observed that 1,8-cineole strikingly reduced the adhesion index of infected erythrocytes to BMEC monolayers. Classically, infected erythrocyte sequestration depends on the recognition of adhesion molecules in the endothelium, such as ICAM-1, and ligands in the infected erythrocytes, such as PfEMP-1. Endothelial adhesion molecules are upregulated during malaria infection and participate not only in parasite sequestration but also in accumulation and recruitment of leukocytes [38], representing a key element in disease pathogenesis. Thus, the decrease in the expression or blockage of such molecules could attenuate susceptibility or severity of the disease. Accordingly, in a murine model of H1N1 infection, treatment with 1,8-cineole inhibited the upregulation of ICAM-1 and VCAM-1 induced by infection, corroborating the anti-inflammatory effect of the compound [39]. Another attractive explanation for the reduction in the adhesion index is impairment of PfEMP1 expression in the surface of infected erythrocytes. This phenomenon could be a result of the direct effect of 1,8-cineol on the erythrocytic cycle of the parasite. However, more experiments are necessary to confirm these hypotheses.

The molecular mechanisms involved in the development of human disease remain to be fully determined, but it is well known that the overall process depends on parasite factors as well as host responses [28, 40]. Interaction among infected erythrocytes, leukocytes, and endothelial cells induces upregulation of proinflammatory cytokines, which leads to activation of brain endothelium [28, 40]. The host immune response and mechanical occlusion culminate in disruption of cerebral blood flow and ultimately lead to dysfunction of the blood-brain barrier, and consequently to hemorrhagic lesions and brain edema [28, 40]. Although the pathology of brain disease in mice seems to be different from that in humans, the use of the murine model of cerebral malaria is well established, and it is considered a valuable tool to study the human disease [41–44]. Using the ECM model, we observed that 1,8-cineole, administered daily right after infection, had anti-plasmodial activity in vivo by decreasing parasitemia consistently until day 6 post-infection. Moreover, at this time point, we observed that the treatment also attenuated the formation of edema. Ramazani et al. [45] demonstrated that extracts of 2 species of *Artemisia* had antiplasmodial activity in BALB/c mice infected with *P*. *berghei*. In their work, the authors could not detect artemisinin in all plants, which suggests the effect of any essential oil constituent present in high amounts in their extracts.

How effectively 1,8-cineole can control the development of cerebral malaria compared to established therapy? Here, we showed that 1,8-cineole attenuated the parasitemia and brain edema. Different from 1,8-cineole treatment, in the presence of artesunate, parasitemia was 100% controlled culminating in the absence of brain edema. These observations agree with studies showing that treatment of infected mice with artesunate (starting right at infection or starting after increased parasitemia), via different administration routes, promote rapid depletion of parasites and effectively attenuate brain inflammation by decreasing leukocytes recruitment [46–48]. If a combination between 1,8-cineole and artesunate could positively cooperate to rescue brain tissue and cognitive function, further experiments will clarify this issue.

The bioavailability of 1,8-cineole is an important factor. McLean et al. [49] has studied the pharmacokinetics of 1,8-cineole on *Trichosurus vulpecula*. The authors showed that intravenous administration of 1,8-cineole revealed it was widely distributed, suggesting the terpene is greatly taken up by tissues. Moreover, about 40% of cineole was eliminated during the distribution phase. On the other hand, when the terpene was administered orally, they observed low bioavailability (at low doses, including 100 mg/kg) due to extensive first-pass metabolism. Moreover, intravenous infusion of cineole induced depression of the central nervous system. However, at the lower blood concentrations caused by oral doses, this undesired effect was not observed. In the present study, we used intraperitoneal administration of 1,8-cineole following the same route of administration used by Murata et al. [50]. We observed a protective effect on cerebral edema caused by malaria infection. In our experiments, 1,8-cineole alone did not change the extravasation of Evens blue dye compared with control mice, suggesting that the terpene administered by this route did not interfere in vascular permeability. However, further experiments are necessary to evaluate the correlation between the administration route and the final effects. Which is the best administration route? The one that demonstrates a good balance between therapeutic and side effects.

The current treatment determined by the World Health Organization is the use of artemisinin analogs in combination with other drugs (ACT) in an attempt to avoid resistance [5]. However, the resistance mechanisms of both *Plasmodium* sp. and malaria vectors to antimalarial drugs and insecticides, respectively, make disease control and extermination extremely difficult [5, 6]. For this reason, a reappraisal of current therapies is indicated, and the discovery of new antimalarial agents is urgently needed. Our results bring new perspectives to the development of innovative therapies to halt malaria disease.

## Supporting information

S1 Fig 1,8-cineole did not induce hemolytic activity. Non-infected erythrocytes (50% hematocrit) were incubated with different concentrations of 1,8-cineole (ranging from 65 to 6483 μM) or 0.5% DMSO (used as vehicle) for 24 h. The hemolytic activity was assessed by measuring free hemoglobin in the cell supernatant as described in the Materials and methods section (*n* = 7). The results are presented as the mean ± SD. n.s., not significant.
(TIF)

S2 Fig Treatment with 1,8-cineole did not change the viability of brain microvascular endothelial cells. BMEC monolayers were incubated with different concentrations of 1,8-cineole (ranging from 324 μM to 3241 μM) for 24 h at 37°C in 5% CO_2_. The cell supernatant was assayed for LDH activity to verify cell viability. The activity was determined as the percentage of a control prepared by adding 1% Triton X-100 to the monolayer (*n* = 4). The results are presented as the mean ± SD. n.s., not significant.
(TIF)
