# Supplementary figures and images for: The monoterpene 1,8-cineole prevents cerebral edema in a murine model of severe malaria
Source: PLoS One. 2022 May 12;17(5):e0268347. doi: 10.1371/journal.pone.0268347 (PMC9098050; doi:10.1371/journal.pone.0268347)

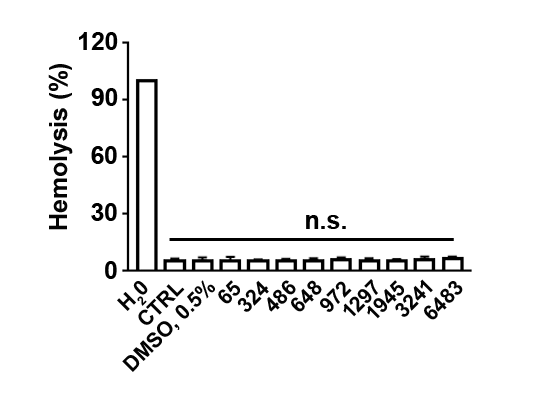

Supplement: S1 Fig — Non-infected erythrocytes (50% hematocrit) were incubated with different concentrations of 1,8-cineole (ranging from 65 to 6483 μM) or 0.5% DMSO (used as vehicle) for 24 h. The hemolytic activity was assessed by measuring free hemoglobin in the cell supernatant as described in the Materials and methods section (n = 7). The results are presented as the mean ± SD. n.s., not significant. (TIF) [file pone.0268347.s001.tif]

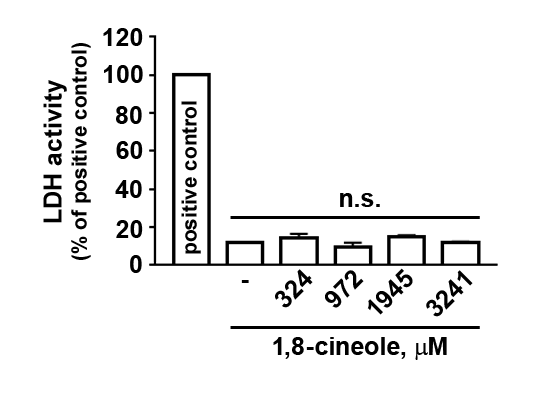

Supplement: S2 Fig — BMEC monolayers were incubated with different concentrations of 1,8-cineole (ranging from 324 μM to 3241 μM) for 24 h at 37°C in 5% CO2. The cell supernatant was assayed for LDH activity to verify cell viability. The activity was determined as the percentage of a control prepared by adding 1% Triton X-100 to the monolayer (n = 4). The results are presented as the mean ± SD. n.s., not significant. (TIF) [file pone.0268347.s002.tif]
